# High Selectivity, Low Damage ICP Etching of *p*-GaN over AlGaN for Normally-off *p*-GaN HEMTs Application

**DOI:** 10.3390/mi13040589

**Published:** 2022-04-09

**Authors:** Penghao Zhang, Luyu Wang, Kaiyue Zhu, Yannan Yang, Rong Fan, Maolin Pan, Saisheng Xu, Min Xu, Chen Wang, Chunlei Wu, David Wei Zhang

**Affiliations:** 1 State Key Laboratory of ASIC and System, School of Microelectronics, Fudan University, Shanghai 200433, China; phzhang19@fudan.edu.cn (P.Z.); wangly20@fudan.edu.cn (L.W.); yangyn20@fudan.edu.cn (Y.Y.); 20212020129@fudan.edu.cn (R.F.); 21112020100@m.fudan.edu.cn (M.P.); ssxu@fudan.edu.cn (S.X.); wuchunlei@fudan.edu.cn (C.W.); 2 Department of Electrical and Electronic Engineering, Xi’an Jiaotong-Liverpool University, Suzhou 215123, China; kaiyue.zhu18@student.xjtlu.edu.cn

**Keywords:** *p*-GaN, selective etching, ICP, surface morphology, MIS capacitor

## Abstract

A systematic study of the selective etching of *p*-GaN over AlGaN was carried out using a BCl_3_/SF_6_ inductively coupled plasma (ICP) process. Compared to similar chemistry, a record high etch selectivity of 41:1 with a *p*-GaN etch rate of 3.4 nm/min was realized by optimizing the SF_6_ concentration, chamber pressure, ICP and bias power. The surface morphology after *p*-GaN etching was characterized by AFM for both selective and nonselective processes, showing the exposed AlGaN surface RMS values of 0.43 nm and 0.99 nm, respectively. MIS-capacitor devices fabricated on the AlGaN surface with ALD-Al_2_O_3_ as the gate dielectric after *p*-GaN etch showed the significant benefit of BCl_3_/SF_6_ selective etch process.

## 1. Introduction

GaN-based high electron mobility transistors (HEMTs) have recently attracted much attention in applications for power switching due to their properties of high-frequency and low on-resistance [1,2]. Two-dimensional-electron-gas (2DEG) is induced by the strong spontaneous and piezoelectric polarization effect in the AlGaN/GaN heterojunction [3], which causes the conventional devices normally be on, i.e., depletion-mode. However, normally-off, i.e., enhanced-mode transistors with a positive threshold voltage are more desirable for simplified circuit design in practice [4,5]. To deplete the 2DEG under the gate area, several approaches have been invented, such as fluorine-implanted treatment [6], gate-recessed [7] and *p*-GaN gate structure [8]. Among these technologies, *p*-GaN gate HEMTs show broad market prospects [9,10].

Precise etch depth control of the *p*-GaN layer with minimum etch damage to the underlying AlGaN barrier is necessary to recover high-density electrons in the access regions, which is the most critical process in the fabrication of *p*-GaN gate HEMTs [11]. Generally, to fully deplete the 2DEG in the channel for normally off operation, a thick *p*-GaN layer with a thin AlGaN layer in epitaxy technique is employed. Further thinning of the AlGaN barrier due to overetching, even a few nanometers, could lead to a dramatic reduction in the conductivity in the access region, which means degradation of the output performance of the devices [12]. On the other hand, an underetched Mg-doped *p*-GaN layer could form a conducting channel contributing to off-state leakage [13]. Therefore, the precise control of *p*-GaN etch depth with minimum damage on AlGaN surface is needed for higher performance E-mode HEMT devices with higher drive current, lower off-leakage and improved dynamic on-resistance [14].

As reported in reference [15], adding SF_6_ gas to BCl_3_ gas would form an AlF_x_ nonvolatile layer on the surface of AlGaN layer after GaN removal, thus achieving high selectivity between GaN and AlGaN, as of 23:1. However, the detailed process optimization and the corresponding impact on the etch damage of AlGaN surface have not been studied yet. In this work, a highly selective ICP etching of *p*-GaN over AlGaN by the BCl_3_/SF_6_ mixture was systematically investigated. The influence of chamber pressure, SF_6_ gas flow, ICP power and bias power on the etch rates and selectivity were studied. The highest selectivity was obtained through process optimization for BCl_3_/SF_6_ etch ambient. Atomic force microscope (AFM) image of AlGaN layer exposed after *p*-GaN selective etching showed a very smooth surface. C–V measurements for Ni/Al_2_O_3_/AlGaN stack MIS structure further confirmed the advantage of this high selective etch process and the minimum etch damage on AlGaN surface.

## 2. Experimental

In this work, two commercially available *p*-GaN/AlGaN/GaN and AlGaN/GaN hetero-structures epitaxially grown on 6-inch Si substrate were used. One is *p*-GaN (80 nm)/Al_0.25_Ga_0.75_N (15 nm)/unintentionally doped GaN (300 nm)/buffer (4.2 μm)/Si (1 mm), and the other is Al_0.25_Ga_0.75_N (15 nm)/unintentionally doped GaN (300 nm)/buffer (4.2 μm)/Si (1 mm). They are referred to *p*-GaN sample and AlGaN sample in the rest of this article.

The etch chamber used to develop the selective *p*-GaN etch process is a customized ICP tool from NAURA (NAURA Technology Group Co., Ltd., Beijing, China) with designed bias power as low as 5 W. For all the processes, a pure BC1_3_ plasma pre-etching was carried out to punch through the (Al)GaN native oxide on the exposed surface [16], right before the main BC1_3_/SF_6_ etch.

The frequencies of power generator and chamber chiller temperature were set as 13.56 MHz and 20 °C, respectively. Etch process conditions were optimized with SF_6_ concentration in the range of 0–30% (constant total flow of 150 sccm), chamber pressure in the range of 20–60 mTorr, ICP power in the range of 200–600 W and bias power in the range of 20–80 W. *p*-GaN and AlGaN samples were etched simultaneously for process evaluation. The etch depth and surface morphology were evaluated using a Park NX10 AFM. Z scanner resolution of this AFM reached 0.015 nm in order that etch depth of patterned samples could be precisely characterized. Etching profiles were inspected by scanning electron microscopy (SEM), and selectivity was calculated as the ratio of the etch rate of *p*-GaN to AlGaN.

## 3. Results and Discussion

### 3.1. Etching Parameter Optimization

#### 3.1.1. SF_6_ Concentration

**Figure 1:**
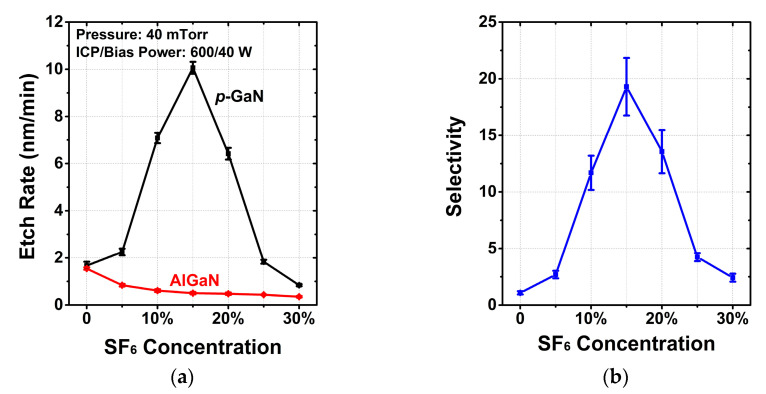
Dependence of (**a**) the etch rates, (**b**) selectivity between *p*-GaN and AlGaN on SF_6_ concentration.

The selective etch process has a strong dependence on the SF_6_ concentration in the ambient, as presented in Figure 1. The other etching conditions were fixed as follows: chamber pressure of 40 mTorr, ICP power of 600 W and bias power of 40 W. A significant enhancement in the *p*-GaN etch rate is observed when the SF_6_ concentration increases from 0 to 15% due to the catalyzed generation of the active chlorine [17,18]. However, further increasing SF_6_ gas flow leads to a decrease in the *p*-GaN etch rate due to the formation of involatile GaF_x_ [18,19]. In summary, adding SF_6_ has two-side impacts on the etch of *p*-GaN and the concentration could be optimized to have the best *p*-GaN etch. For the AlGaN sample, the etch rate monotonically decreases with increasing SF_6_ concentration due to the formation of nonvolatile AlF_x_ acting as a powerful etch-stop layer [15]. The selectivity reaches a maximum at 15% SF_6_.

#### 3.1.2. Chamber Pressure

**Figure 2:**
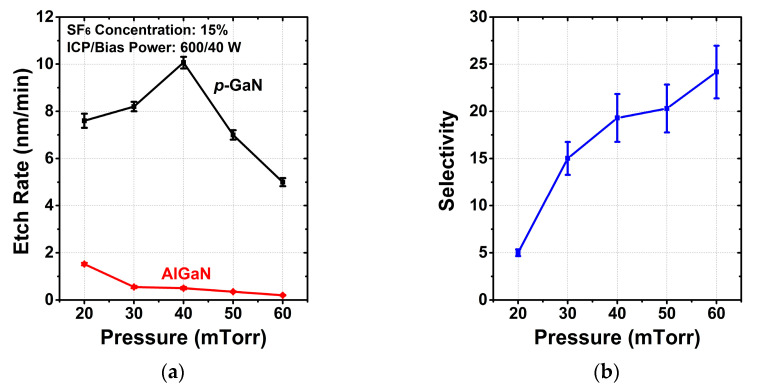
Dependence of (**a**) the etch rates, (**b**) selectivity between *p*-GaN and AlGaN on chamber pressure.

The effects of chamber pressure on the etch rates of *p*-GaN and AlGaN layers and the selectivity were examined, as shown in Figure 2. The other etching conditions were fixed as follows: SF_6_ concentration of 15%, ICP power of 600 W and bias power of 40 W. At the beginning of increasing chamber pressure, more chemical radicals of chlorine are generated to react with *p*-GaN in order that the etch rate keeps increasing. When the pressure is higher than 40 mTorr, the formed involatile GaF_x_ becomes the dominant to suppress the etching of *p*-GaN. For AlGaN, more AlF_x_ will be formed with higher pressure to reduce the etch rate. The etch selectivity increases from 5:1 to 24:1 as the chamber pressure increases from 20 to 60 mTorr. However, there is a trade-off in the pressure range of 40–60 mTorr, considering the slight improvement in selectivity and the sharp drop of *p*-GaN etch rate.

#### 3.1.3. ICP Power

The ICP etching mechanism is a combined process of chemical reaction and ion sputtering. Both plasma density and ion energy can be regulated independently with two RF generators, i.e., ICP and bias power generators. Thus, the variation of source power and bias power can effectively affect the proportion of chemical and physical etching.

**Figure 3:**
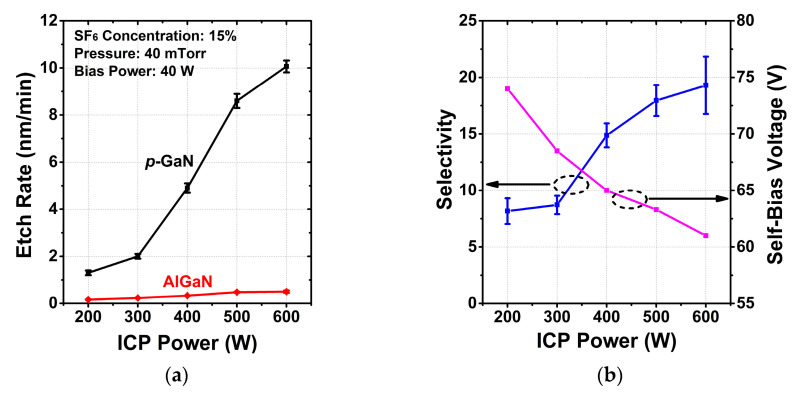
Dependence of (**a**) the etch rates, (**b**) selectivity between *p*-GaN and AlGaN and self-bias voltage on ICP power.

The etch rates, selectivity and self-bias voltage as a function of ICP power are shown in Figure 3. The other etching conditions were fixed as follows: SF_6_ concentration of 15%, pressure of 40 mTorr and bias power of 40 W. The plasma density and fractional ionization are controlled by the ICP power. The etch rate of the *p*-GaN sample remarkably increases from 1.3 to 10.1 nm/min with increasing ICP power from 200 to 600 W due to more activity and density of the chemical radicals. The declining self-bias voltage is associated with increasing plasma density, indicating that the plasma bombardment is weakened. Increasing chemical reaction proportion and decreasing physical bombardment are exactly desired for high selectivity and low etch damage. However, the AlGaN etch rate slightly increases owing to the competition between chlorine as the etching agent and fluorine as the inhibition agent. AlF_x_ can be formed more easily than GaF_x_, preventing a quick increase of AlGaN etching. As a result, the *p*-GaN/AlGaN etch selectivity increases with ICP power.

#### 3.1.4. Bias Power

**Figure 4:**
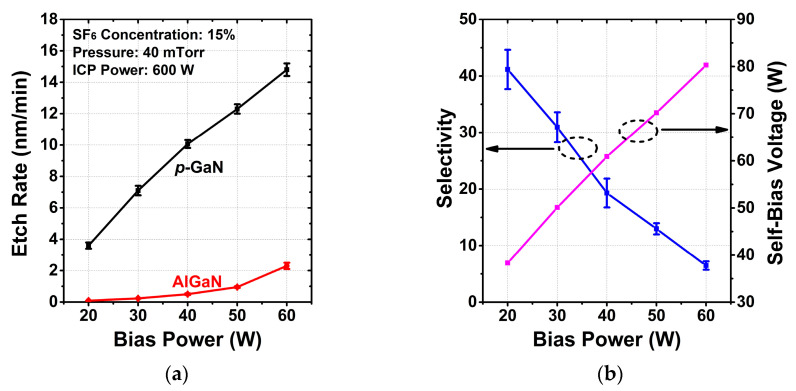
Dependence of (**a**) the etch rates, (**b**) selectivity between *p*-GaN and AlGaN and self-bias voltage on bias power.

The effects of bias power are shown in Figure 4. The other etching conditions were fixed as follows: SF_6_ concentration of 15%, pressure of 40 mTorr and ICP power of 600 W. The bias power is strongly related to physical etching. The self-bias voltage decreases linearly as the bias power decreases, indicating reduced ion bombardment energy. Though both *p*-GaN and AlGaN etch rates decrease proportionally to the decreasing bias power, almost linearly increasing selectivity is obtained. When the bias power drops down to 20 W, the selectivity reaches a maximum of 41:1 at a *p*-GaN etch rate of 3.4 nm/min in this study. The reduction in selectivity at high bias power can be explained in terms of enhanced sputtering of the AlF_x_ film at the AlGaN surface.

To sum up, the final optimized process conditions were determined as SF_6_ concentration of 15%, chamber pressure of 40 mTorr, ICP power of 600 W and bias power of 20 W. Table 1 summarizes the results achieved in our work together with other research using the BCl_3_/SF_6_ mixture. The selectivity in this study is the highest value ever reported, which can be attributed to our systematic optimization and the lowest bias power of our designed etch tool. Additionally, as reported in reference [20], a high selectivity of 33:1 was realized by using a higher frequency bias generator of 40 MHz. The much higher plasma frequency produces lower-energy ions which tends to achieve higher selectivity but with much lower etch rate. This makes the developed process in this work more practical in real device fabrication.

### 3.2. Etched Surface and Plasma Damage Analysis

**Figure 5:**
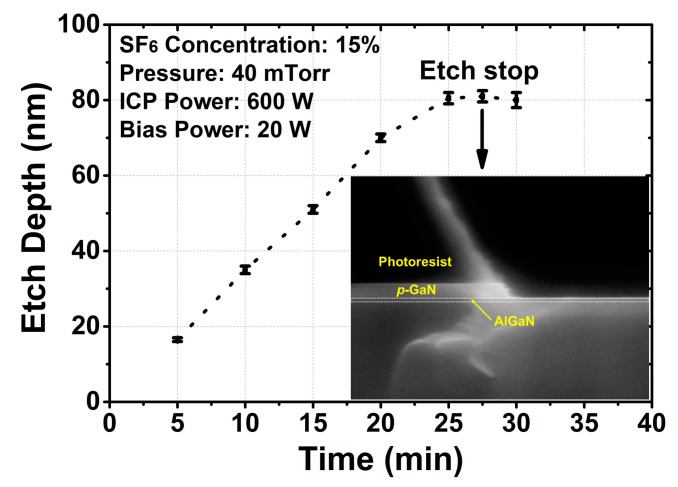
*p*-GaN etch depth as a function of time; the inset X-SEM image of sample with 2.5 min of overetching.

To comprehensively study the practical effects of the developed process on *p*-GaN/AlGaN wafer, firstly the etch depth was measured at different etch times by AFM. As seen in Figure 5, the etch process was quite linear until it reached the AlGaN surface. The X-SEM in the inset clearly shows a very smooth and almost non-recessed AlGaN surface after 2.5 min of overetching under the optimized process, demonstrating a highly selective etch to the AlGaN layer.

To further evaluate the impact of the developed selective etching process on the AlGaN surface, AFM images (5 μm × 5 μm) of the surface morphology were taken in no-contact mode (NCM) for the abovementioned sample (sample A, 2.5 min over etching under the optimized process) and another etched sample by using the nonselective BCl_3_/Ar process (sample B) to etch the 80 nm *p*-GaN layer. The nonselective process has a *p*-GaN etch rate of approximately 10 nm/min.

**Figure 6:**
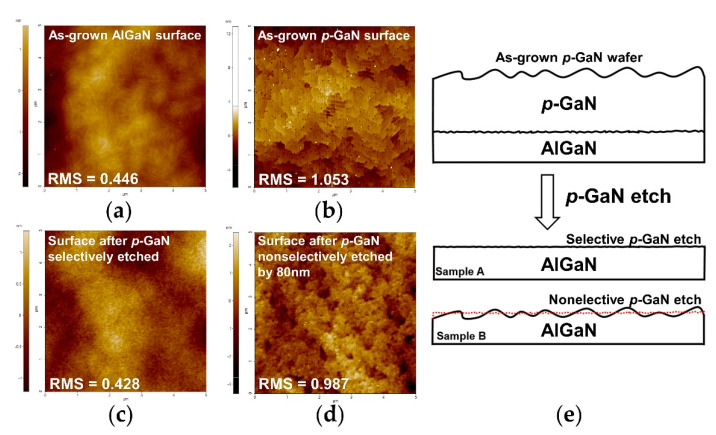
Surface morphology of (**a**) as-grown AlGaN surface, (**b**) as-grown *p*-GaN surface, (**c**) sample A (*p*-GaN selectively etched) and (**d**) sample B (*p*-GaN nonselectively etched), (**e**) diagram to illustrate surface morphology for samples A and B.

As seen in Figure 6, for sample A the exposed AlGaN surface is quite smooth with the root mean square (RMS) surface roughness of 0.428 nm, which is similar to the as-grown AlGaN surface (0.446 nm in Figure 6a). This is attributed to the advantage of the developed highly selective etching and its low power causing very minimum surface damage. However, with nonselective *p*-GaN etching for sample B, the exposed AlGaN surface roughness reached as high as 0.987 nm. This is equivalent to the as-grown *p*-GaN surface, which has 1.053 nm RMS roughness due to the doping of Cp_2_Mg. Obviously, the sample B AlGaN surface is much rougher as the morphology is basically inherited from the as-grown *p*-GaN layer due to the nature of nonselective etching, as illustrated in Figure 6e.

MIS capacitors were fabricated to evaluate the possible etch damage on the exposed AlGaN surface for samples A and B. Reference device on as-grown AlGaN wafer was also prepared for comparison. For all the samples, dilute HCl dip was performed to treat the AlGaN surfaces, and 25-nm-thick Al_2_O_3_ was deposited at 300 °C using trimethylaluminum (TMA) and H_2_O as precursors. Ni/Au bilayers were used as electrodes in the inner circle and exterior zone with a ring gap of 50 μm. To avoid the possible repair effect on the etch damage, no anneal was performed in this process.

**Figure 7:**
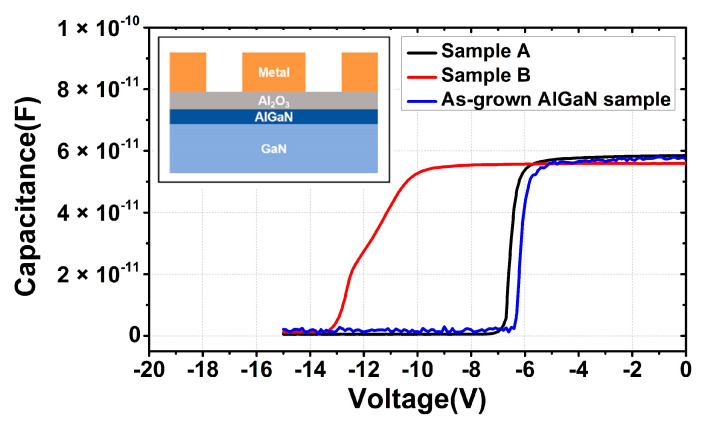
C–V characteristics of Ni/Al_2_O_3_/AlGaN MIS capacitors with selective and nonselective *p*-GaN etching. Additionally, as-grown AlGaN sample is referenced here.

C–V characterizations using a Keythley 4200A are presented in Figure 7. At a quite negative voltage, the capacitance is close to zero because the 2DEG is depleted for all samples. The nearly flat capacitance C_2DEG_ indicates that 2DEG has been formed at the AlGaN/GaN heterojunction interface. For sample A with selective *p*-GaN etching, the slope of the C–V curve is quite steep and close to the reference as-grown AlGaN sample, confirming very minimum etch damage on the AlGaN surface after selective *p*-GaN removal. However, the slope of the C–V curve for sample B shows an obvious stretch-out, indicating that the exposed AlGaN barrier layer was degraded during the nonselective etching of *p*-GaN and thus 2DEG at AlGaN/GaN interface could not be formed efficiently with the gate bias.

## 4. Conclusions

In this work, a highly selective ICP etch process of *p*-GaN over AlGaN using the BCl_3_/SF_6_ mixture was successfully developed, achieving a high selectivity of 41:1. Under the conditions of the optimized SF_6_ concentration and chamber pressure, as well as high ICP power, and as low as possible bias power benefiting from our dedicated etch tool, a very smooth and high-quality AlGaN surface could be obtained after *p*-GaN etch. On such AlGaN surface, the fabricated Ni/Al_2_O_3_/AlGaN MIS capacitor showed comparable C–V characteristics to that on the as-epitaxial AlGaN surface. This phenomenon strongly indicated that there was almost no damage on the AlGaN surface after etching the *p*-GaN layer, making this process very promising to be applied on the fabrication of high-performance *p*-GaN gate HEMTs.

## Figures and Tables

**Table 1:** Comparison of etch conditions, etch rates and selectivity among BCl_3_/SF_6_ mixture selective etching recipes.

Reference	[15]	[21]	This Work	[20]
Generator (MHz)	13.56	13.56	13.56	40
SF_6_%	20	40	15	40
Pressure (mTorr)	37.5	20	40	10
ICP power (W)	200	200	600	/
Bias power (W)	30	60	20	/
GaN etch rate (nm/min)	12	12	3.4	0.529
AlGaN etch rate (nm/min)	0.52	1.3	0.08	0.016
Max. selectivity	23:1	9:1	41:1	33:1

## Data Availability

Not applicable.
